# Gas Dynamic Virtual Nozzle Sprayer for an Introduction of Liquid Samples in Atmospheric Pressure Ionization Mass Spectrometry

**DOI:** 10.1021/acs.analchem.2c05349

**Published:** 2023-02-17

**Authors:** Barbora Kloudová, Timotej Strmeň, Vladimír Vrkoslav, Zdeněk Chára, Ondřej Pačes, Josef Cvačka

**Affiliations:** † Institute of Organic Chemistry and Biochemistry of the Czech Academy of Sciences, Flemingovo nám. 2, CZ-166 10 Prague 6, Czech Republic; ‡ Department of Analytical Chemistry, Faculty of Science, Charles University in Prague, Hlavova 2030/8, CZ-128 43 Prague 2, Czech Republic; § Institute of Hydrodynamics of the Czech Academy of Sciences, Pod Pat’ankou 30/5, CZ-166 12 Prague 6, Czech Republic

## Abstract

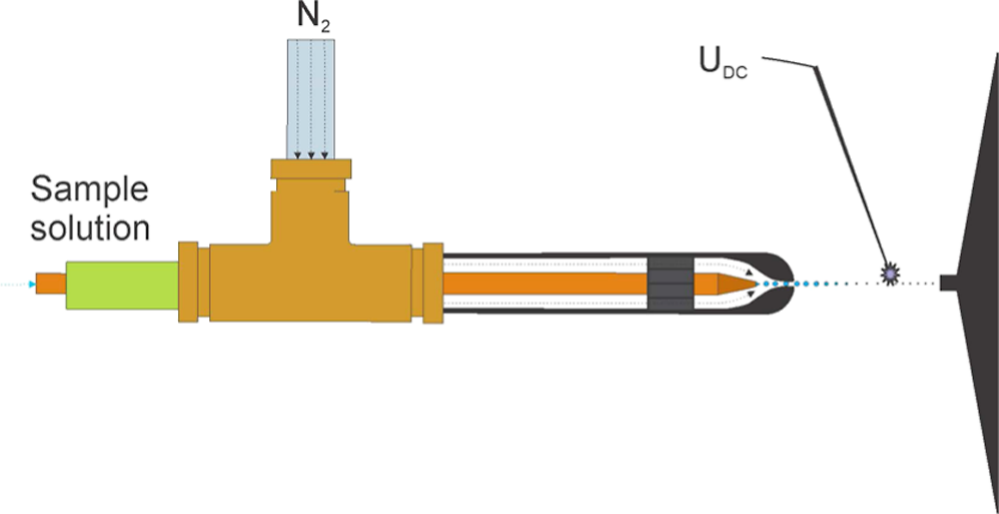

Electrospray may exhibit inadequate ionization efficiency in some applications. In such cases, atmospheric-pressure chemical ionization (APCI) and photoionization (APPI) can be used. Despite a wide application potential, no APCI and APPI sources dedicated to very low sample flow rates exist on the market. Since the ion source performance depends on the transfer of analytes from the liquid to the gas phase, a nebulizer is a critical component of an ion source. Here, we report on the nebulizer with a gas dynamic virtual nozzle (GDVN) and its applicability in APCI at microliter-per-minute flow rates. Nebulizers differing by geometrical parameters were fabricated and characterized regarding the jet breakup regime, droplet size, droplet velocity, and spray angle for liquid flow rates of 0.75–15.0 μL/min. A micro-APCI source with the GDVN nebulizer behaved as a mass-flow-sensitive detector and provided stable and intense analyte signals. Compared to a classical APCI source, an order of magnitude lower detection limit for verapamil was achieved. Mass spectra recorded with the nebulizer in dripping and jetting modes were almost identical and did not differ from normal APCI spectra. Clogging never occurred during the experiments, indicating the high robustness of the nebulizer. Low-flow-rate APCI and APPI sources with a GDVN sprayer promise new applications for low- and medium-polar analytes.

## Introduction

Detection of organic compounds using atmospheric pressure ionization (API) mass spectrometry is of paramount importance in modern liquid chromatography. A common feature of these techniques is the formation of an aerosol from a liquid sample at atmospheric pressure in the ion source.^1,2^ While electrospray generates charged droplets, nebulizers in atmospheric-pressure chemical ionization (APCI) and photoionization (APPI) sources atomize liquids to neutral particles. Ionization in APCI and APPI occurs in the gas phase after solvent evaporation from the droplets.^3^ Although electrospray ionization (ESI) works well for most liquid chromatography/mass spectrometry (LC/MS) applications, it may exhibit low ionization efficiency for less-polar and nonpolar compounds. APCI and APPI techniques are suited well for such substances.^4^ Compared to ESI, APCI and APPI suffer less from matrix effects and accept a wider range of solvents,^5,6^ making the techniques attractive for the sensitive detection of metabolites, natural products, drugs, pesticides, and other compounds in classical and omic applications.^4−10^

APCI, developed in the 1970s, represented the first ion source working at atmospheric pressure. The original ^63^Ni source of electrons has been replaced by a corona discharge electrode.^11,12^ APCI began to gain importance in the early and mid-1980s when the ion source performance was significantly improved by using a direct liquid introduction probe,^13^ followed by a heated pneumatic nebulizer.^14^ The heated pneumatic nebulizer interface has been used in commercial instruments since that time. The liquid sample flowing out of a capillary in a heated tube is nebulized into small droplets. Rapid vaporization ensures that the analyte molecules are not decomposed. The solvent and sample vapors mixed with the nebulizing gas continue to the corona discharge region, where ionization takes place.^1,15,16^

Over the decades, significant progress has also been made in chromatography. The need for higher sensitivity, lower detection limits, and shorter and cheaper analyses resulted in the miniaturization of separation systems.^17^ The inner column diameters were reduced to achieve high-performance liquid chromatography (HPLC) in capillary and nanoformats. Scaling down the HPLC separations requires detectors optimized for low-mobile-phase flow rates. ESI is easy to operate at reduced flow rates, and commercial ion sources for micro- and nano-ESI are readily available. In contrast to ESI, an APCI source for microliter-per-minute or even lower flow rates has not been commercialized yet. It can be argued that APCI-MS is a mass-sensitive detector, which may cause reduced sensitivity at low flow rates. However, ESI-MS, a concentration-sensitive detector, also behaves as a mass-sensitive device at nanoliter-per-minute flow rates.^18^ Therefore, it is likely to achieve high detection sensitivity also in APCI at low flow rates, if efficient ionization and ion transfer to the mass analyzer are ensured. Several works have been devoted to the design of the APCI sources for low flow rates.^17,19−23^ The first type was a microheated nebulizer interface assembled from three concentrically arranged fused silica capillaries, serving for sample, nebulizing, and auxiliary gas delivery. The nebulizer was optimized for flow rates of 0.15–1.6 μL/min and demonstrated good linearity and a low detection limit.^20^ The subsequent development relied on modifications of commercial high-flow platforms. The inlet capillary was replaced with two concentrically positioned capillaries for the sample supply and delivery of nebulizing gas. The modified APCI interfaces for coupling with capillary electrophoresis and micro-HPLC were operated at 1–10 μL/min.^19,24^ A microchip-based nebulizer was developed several years later. It consisted of anodically bonded glass and silicon plates, with an aluminum heater sputtered on the glass surface. A channel etched in the silicon wafer delivered the sample and nebulizing gas. The microchip APCI source provided good repeatability and linearity of analyte signals^17^ and allowed the detection of LC-separated analytes like neurosteroids.^25^ Later, the chip was modified to an all-glass version with a platinum heater,^26^ enabling the analysis of less-volatile analytes, including neutral lipids.^21^ Another micro-APCI source was developed and tested in our laboratory.^22^ Its nebulizer was assembled from a corundum or quartz tube on which a resistance wire was wound and a fused-silica capillary was inserted into it. Such a nebulizer was easy to make. However, its performance was limited by its susceptibility to clogging and memory effects manifested by tailing chromatographic peaks.

The performance of an APCI or APPI ion source depends largely on the transport efficiency of analyte molecules from the liquid to the gas phase. The nebulizer plays a key role in this process because it determines the size and velocity distribution of the droplets, spray divergence, and stability. Understanding the physical processes during liquid spraying at low flow rates can help us design efficient nebulizers for low-flow APCI or APPI. As follows from the seminal research of Rayleigh in the 1800s, any free cylindrical jet of liquid emerging in the laminar flow from an orifice breaks up spontaneously to form a train of spherical droplets.^27^ Spontaneous breakup yields a narrow distribution of droplets, and small droplets can be generated simply by reducing the orifice diameter. However, nozzles with a diameter lower than 10 μm are prone to clogging, preventing the formation of droplets smaller than ∼20 μm.^28^ This limitation can be overcome using a pneumatic flow-focusing nebulizer introduced by Gañán-Calvo.^29^ In his design, a capillary supplied a liquid at microliters per minute flow rates. The mouth of the capillary was positioned close to a small hole in a thin plate through which a gas stream was flowing. A steady thin liquid jet was created, which stretched through the hole for several millimeters, depending on the liquid flow rates and gas velocity.^29^ The coflowing gas created a nozzle that functioned similarly to a solid wall nozzle. The device provided a robust monodisperse spray of micrometer-sized droplets. The original Gañán-Calvo design was modified by DePonte 10 years later by replacing the plate with a 1.2 mm O.D. borosilicate tube with a fire-polished mouth.^28^ Fire processing created an aerodynamic constriction at the end of the tube, yielding a radially symmetric, convergent exit channel. The inner capillary tube was tapered and centered within the borosilicate outer housing. The device was named “gas dynamic virtual nozzle” (GDVN) to emphasize that coaxial gas flow creates an imaginary nozzle that reduces the diameter of the liquid jet in a similar way to a regular nozzle. GDVN has been proven to be a reliable source of well-collimated microscopic droplets. Depending on the flow rate and pressure conditions, GDVN could operate in three principal regimes: (i) dripping, characterized by the emission of relatively large (micrometer size), regularly spaced droplets, (ii) unsteady dripping, and (iii) jetting that yields a continuous stream of small droplets. Unlike standard micronozzles, the GDVN is resistant to clogging. The reliable generation of microscopic droplets and liquid jets delivered by GDVN is used in many applications, one being the crystallography of large biomolecules.^30^ To our knowledge, GDVN has never been used for nebulizing liquid samples in organic mass spectrometry.

In this study, we investigate the utility of GDVN for nebulizing liquid samples at microliter-per-minute flow rates in APCI-MS. A series of GDVN nebulizers differing by the exit channel diameter and capillary-to-exit channel distance were fabricated and investigated under various conditions to evaluate the droplet sizes, velocities, and spray plume shapes. The GDVN sprayers were employed in an in-house, simplified APCI source to demonstrate their ability to create ions from test compounds. Various compounds were detected at flow rates down to 750.0 nL/min. The sprayers provided stable and intense analyte signals. Although the ion source was not equipped with a heater, the GDVN nebulized the liquid samples efficiently. Compared to a classical APCI source, an order of magnitude lower detection limit for verapamil was achieved with the GDVN working in a jetting mode. No sprayer clogging was observed throughout the experiments.

## Experimental Section

### Fabrication of the Nebulizers

The sprayer was manufactured from a borosilicate glass tube (1.50 mm O.D., 1.17 mm I.D., World Precision Instrument, Sarasota, FL) and a tapered tip capillary Sharp Singularity emitter (50 μm I.D., 365 μm O.D.) from Fossil Ion Technology S.L. (Madrid, Spain). To fabricate the GDVN outer housing, one of the ends of the borosilicate glass tube was heated in micropipette puller P 100 (Sutter Instrument, Novato, CA); see Text S1 for details. In contrast to the original work,^28^ the GDVN sprayer was designed as dismountable, making it possible to change the position of the tapered capillary during experiments. The sprayer was assembled using a PEEK Tee connector (0.040″ bore, 1/16″ O.D. tubing, 1/4–28 flat bottom; IDEX Health and Science LLC, Oak Harbor, WA) serving as a junction for the tapered tip capillary, the borosilicate glass outer housing, and a 1/16″ O.D. Teflon tubing for the nebulizing gas. The tapered tip capillary was tightened in the Tee connector using a 1/16″ O.D. fluoropolymer sleeve.

### Hydrodynamic Experiments

The droplets emerging from the GDVN sprayers were observed by the Phantom VEO 410 high-speed camera (Dante Dynamics, Denmark), which operated at a frame rate of 20 kHz with an image size of 1280 × 200 pixels; 3000 frames were taken for each recording. A cylindrical lens line light KL 2500 (Leica Microsystem Germany) was used to illuminate the scanned plane. The data were analyzed in the Matlab program (MathWorks, Natick, MA), where the original images were converted into binary form. Then, the trajectories of individual droplets were monitored, and the instantaneous velocities were found. The frequencies at which the droplets appeared at the orifice were determined. The frequencies were used to calculate the droplet sizes (only for nebulizers operated in dripping regimes).

### Determination of the Spray Angle

The divergence of the droplet stream was estimated from the spots created by spraying a dye solution on filter paper. The experiments were performed with nebulizers N2–N4 operated in dripping and jetting modes and positioned perpendicularly to the filter paper at a distance of 0.5–4.0 cm. Coomassie dye in methanol (0.1 mmol/L) was sprayed at a flow rate of 1.0 μL/min for 45 s. The blue spots were scanned, and the images were processed in ImageJ software (National Institute of Health, NY) to generate density profile plots. The peak widths at the baseline were used to estimate the spray plume diameter at a given distance. The spray angle Θ was calculated from the linear fit of data points representing dye spot radius at various spraying distances. The arctangents of the slope of the fitted line equaled the spray half-angle, which was multiplied by 2 to get the spray angle Θ.

### Micro-APCI Source

The ion source was assembled on a platform consisting of a flange with two guiding rods. The rods supported a micromanipulator MX10r (Siskiyou, Grant Pass, OR) with the GDVN sprayer and a holder for the corona discharge needle. The needle tip was placed at a distance of 5.0 mm from the inlet capillary and 2.0 mm from the GDVN orifice; the tip was positioned slightly off-axis not to shield the inlet (Figure S1). A GFCS-011771 mass flow controller (Aalborg, Orangeburg, NY) installed on the instrument nebulizer gas line allowed us to adjust the nitrogen flow rate up to 500 mL/min. The liquid samples were infused at flow rates of 0.75–20.0 μL/min using a NE-300 syringe pump (New Era Pump System Inc., Toledo, NY). No heating element to aid nebulization was used.

### Mass Spectrometry

The mass spectra were acquired using an LCQ Fleet mass spectrometer (Thermo Fisher Scientific, San Jose, CA) equipped with the in-house-made micro-APCI source operated in the positive ion mode with the parameters set as follows: corona discharge current 1.0 μA, capillary temperature 300 °C, capillary voltage 40.0 V, and tube lens 100.2 V. The standard APCI source (Ion Max-S API source from Thermo Fisher Scientific) was operated at the same voltage setting; the other parameters were set as follows: probe vaporizer temperature 400 °C, corona discharge current 5.0 μA, and sheath and auxiliary gas flow rates 40 and 10 arbitrary units, respectively. The calibration solutions of verapamil were prepared in toluene and infused into the micro-APCI source at 1.5 μL/min using a syringe pump. In the case of the standard APCI source, the calibration solutions delivered at 1.5 μL/min were mixed in a T-union with toluene (1.0 mL/min) and infused into the ion source. The calibration curves were constructed from the verapamil signal (*m*/*z* 455.2, [M + H]^+^) obtained by averaging spectra in 1 min record. The GDVN spray stability was tested by direct infusion of verapamil (1.0 μmol/L in toluene) at 1.5 and 4.0 μL/min. The verapamil signal was averaged for approximately 30 s intervals (50 scans) within a 10 min experiment.

## Results and Discussion

### Fabrication of GDVN Nebulizers

**Figure 1:**
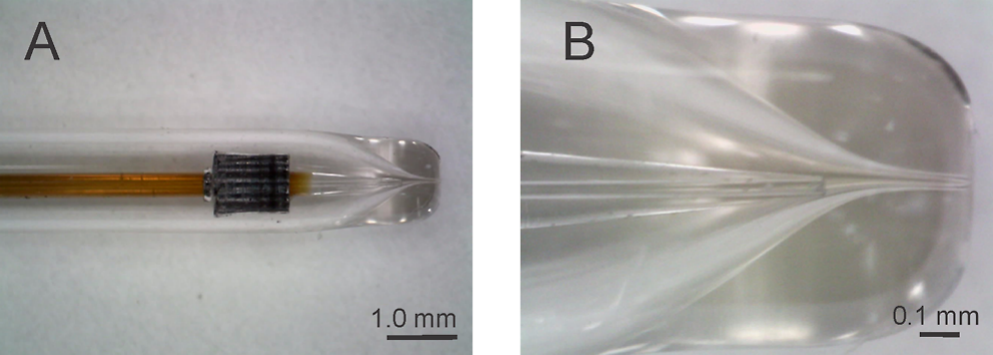
End part of the GDVN nebulizer N1 (A) and a close-up view of the nozzle (B).

The design of the nebulizers was based on the work of DePonte.^28^ After testing several procedures, the most reproducible fabrication of the outer glass housing was achieved with a micropipette puller (Text S1). The PTFE sleeve used in the original work^28^ for centering the fused silica capillary inside the outer housing was replaced by a small aluminum alloy element which offered significantly less resistance to the flowing gas and reliably kept the capillary in the central axis (Figure 1).

**Figure 2:**
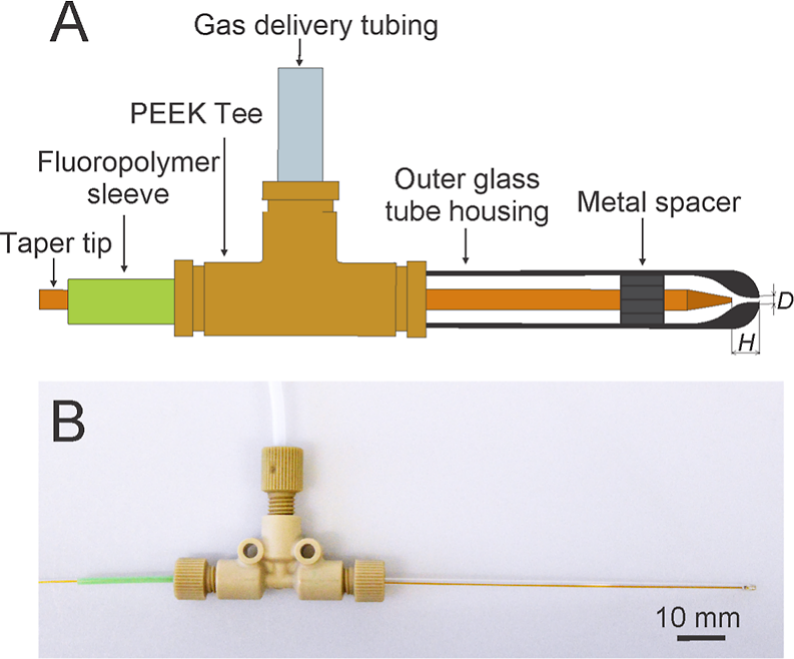
Schematic drawing (A) and a photograph (B) of the dismountable GDVN nebulizer. The drawing shows GDVN geometric parameters: capillary-to-exit channel distance *H* and exit channel diameter *D*.

The GDVN nebulizers used in this work were designed as dismountable, making it possible to change the position of the tapered capillary during experiments (Figures 2 and S2).

A total of seven different nebulizers were manufactured and tested in this work; their overview is given in Table 1.

**Table 1:** List of the Nebulizers and Their Geometrical Parameters

nebulizer	exit channel diameter *D* [μm]	capillary-to-exit channel distance *H* [μm]
N1	47	503
N2	82	470
N3	105	401
N4	155	340
N5	260	320
N6	295	301
N7	320	296

### Effects of GDVN Parameters on the Spray Regime

Droplet formation and working regime depend strongly on geometric parameters such as the diameter of the exit channel in the outer housing (parameter *D* in Figure 2) and the distance of the tapered capillary tip from the exit channel (parameter *H* in Figure 2). It also depends on the nebulizing gas and sample flow rates.^28^ Two regimes of the jet breakup, dripping and jetting, were observed. The dripping occurs when the liquid momentum is not sufficiently large. The meniscus (a liquid portion between the capillary tip and exit channel) breaks up into relatively large droplets. When the liquid flow rate is increased beyond a certain limit, the inertial forces start to dominate the surface tension forces; steady streaming of a liquid jet, that is, jetting, takes place.^31−33^

The breakup regimes were observed after illuminating the jet emerging from a horizontally placed nebulizer with an intense line light (Figure S3). The dripping regime was characterized by a narrow stream of large droplets, leaving the nozzle at regular intervals, one after the other. The droplets traveled several centimeters before their trajectory began to turn toward the ground due to gravity. In contrast, the droplets in the jetting regime were significantly smaller and formed a continuous spray. The transition between the dripping and jetting regimes occurs at a critical Weber number (*We*), which is a measure of the relative importance of the fluid’s inertia compared to its surface tension.^31^ Since *We* = ρ*v*^2^*D*/σ, where ρ is the density of a liquid with velocity *v* through a tube of the diameter *D*, the jet regime is affected by the exit channel diameter. The GDVN nebulizers with an exit channel diameter larger than approximately 200 μm (N5–N7) worked in our conditions exclusively in a dripping mode, characterized by relatively large droplets released in regular intervals. The nebulizers with smaller openings (N2–N4) exhibited either dripping or jetting behavior depending on the gas flow rate. While lower gas flow rates induced the dripping mode, higher settings favored a jetting mode with a conical stray of fine droplets. For example, nebulizers N3 (*D* = 105 μm) and N4 (*D* = 155 μm) discharging water at 1.5 μL/min exhibited a dripping regime at gas flow rates of 50 and 125 mL/min, respectively, and a jetting mode above 80 and 175 mL/min, respectively. The N1 nebulizer (*D* = 47 μm) could not reach the dripping mode even at very low gas flow rates. The transition between the jet breakup regimes occurred at different gas flow rates for N2–N4. The transition between the two regimes was not always clearly visible. A sharp transition is more likely to be achieved in solvents with a higher viscosity than water.^32,34^

### Effects of GDVN Parameters on Droplet Size and Velocity

**Figure 3:**
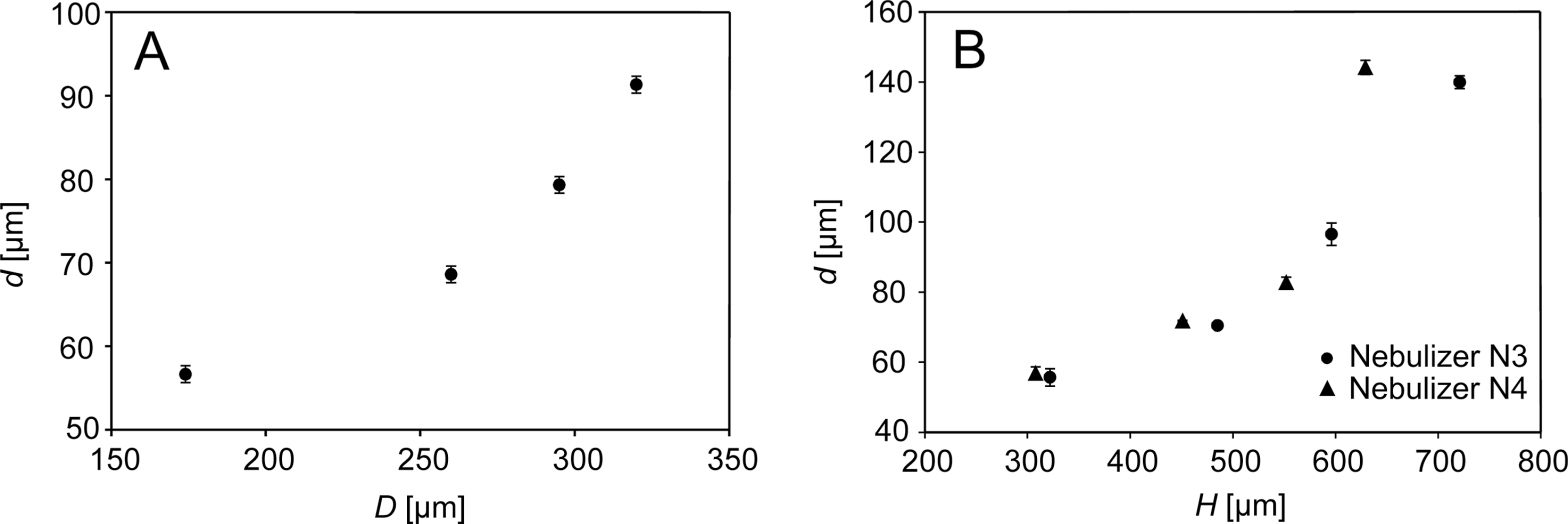
Droplet diameter as a function of the exit channel diameter (A). Data are shown for the nebulizers N4–N7 discharging water at 5.0 μL/min; the dripping mode was achieved at a nitrogen flow rate of 250 mL/min. Droplet diameters as a function of the capillary-to-exit channel distance (B) for the nebulizers N3 and N4 discharging water at 5.0 μL/min; the dripping mode was achieved at a nitrogen flow rate of 55 mL/min (N3) and 90 mL/min (N4). The error bars represent standard deviations (*n* = 3).

A high-speed camera was used to characterize droplets emerging from the GDVN nebulizers. The experiments could only be performed for nebulizers in the dripping mode, generating relatively large droplets. The frequency at which the droplets leave the nebulizer orifice was used to calculate the droplet volume (Text S2). The droplet diameter *d* increased with the exit channel diameter *D* (Figure 3A), which agrees with the theory of the breakup of cylindrical liquid jets discharging into a gas.^35−38^ When nebulizing a liquid sample, it is desirable to create small droplets that evaporate easily. The smallest droplets generated with the nebulizers operated in the dripping regime were about 50 μm in diameter. Such droplets are larger than primary droplets generated by other API methods. For instance, the mean diameter of water droplets generated by the thermospray was estimated to be 10–30 μm.^39^ Depending on experimental conditions, the electrospray provides microdroplets 1–10 μm in diameter,^40,41^ and the initial droplet diameters in nanoflow electrospray are in the hundreds of nanometer range.^42^

The tapered tip capillary was kept in the center axis of the outer glass housing. The dismountable design of the nebulizers allowed us to investigate the effect of the capillary-to-exit channel distance *H* on the droplet size. As shown for the nebulizers N3 and N4 (Figure 3B), the droplet size decreased with decreasing *H*. The capillary-to-exit channel distance affected the gas–liquid interaction angle at the meniscus. With increasing *H*, the gas–liquid interaction angle gets smaller, which causes local viscous forces to be less effective in droplet formation. Droplets get bigger until viscous forces become so weak that any droplet is formed.^43^ In our case, no spray was formed for *H* over 700 μm. Under these conditions, water flowed freely from the capillary and eventually flooded the outer housing. Conversely, the capillary tip positioned very close to the exit channel restricted gas flow through the nozzle, which resulted in increased pressure in the nebulizer. Since the orifice shape was, to some extent, unique for each manually fabricated outer glass tube, the capillary-to-exit channel distance had to be optimized experimentally for each nebulizer unit.

**Figure 4:**
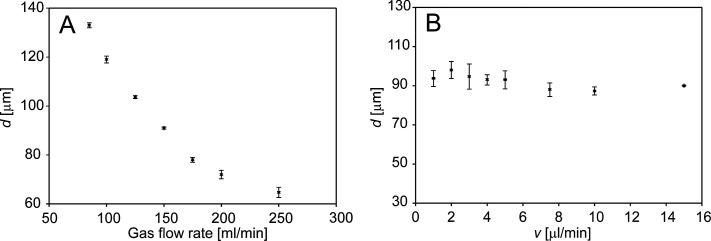
Droplet diameter as a function of the gas flow rate for the nebulizer N5 discharging water at 5.0 μL/min (A). Droplet diameter as a function of the flow rate of water for the same nebulizer at a constant gas flow rate of 150 mL/min (B). The error bars represent standard deviations (*n* = 3).

The effects of gas and liquid flow rates on droplet formation were investigated using the N5 nebulizer operated in the dripping regime. The droplet size decreased with increasing gas flow rate (Figure 4A) but remained almost the same when the liquid flow rate increased (Figure 4B). The liquid jet focusing was much stronger at a higher gas flow rate, which induced a higher pressure drop across the nozzle. The increasing pressure in the nozzle caused the droplet size to decrease. Increasing the liquid flow rate did not change the pressure much, and therefore, the size of the droplets remained almost constant.

**Figure 5:**
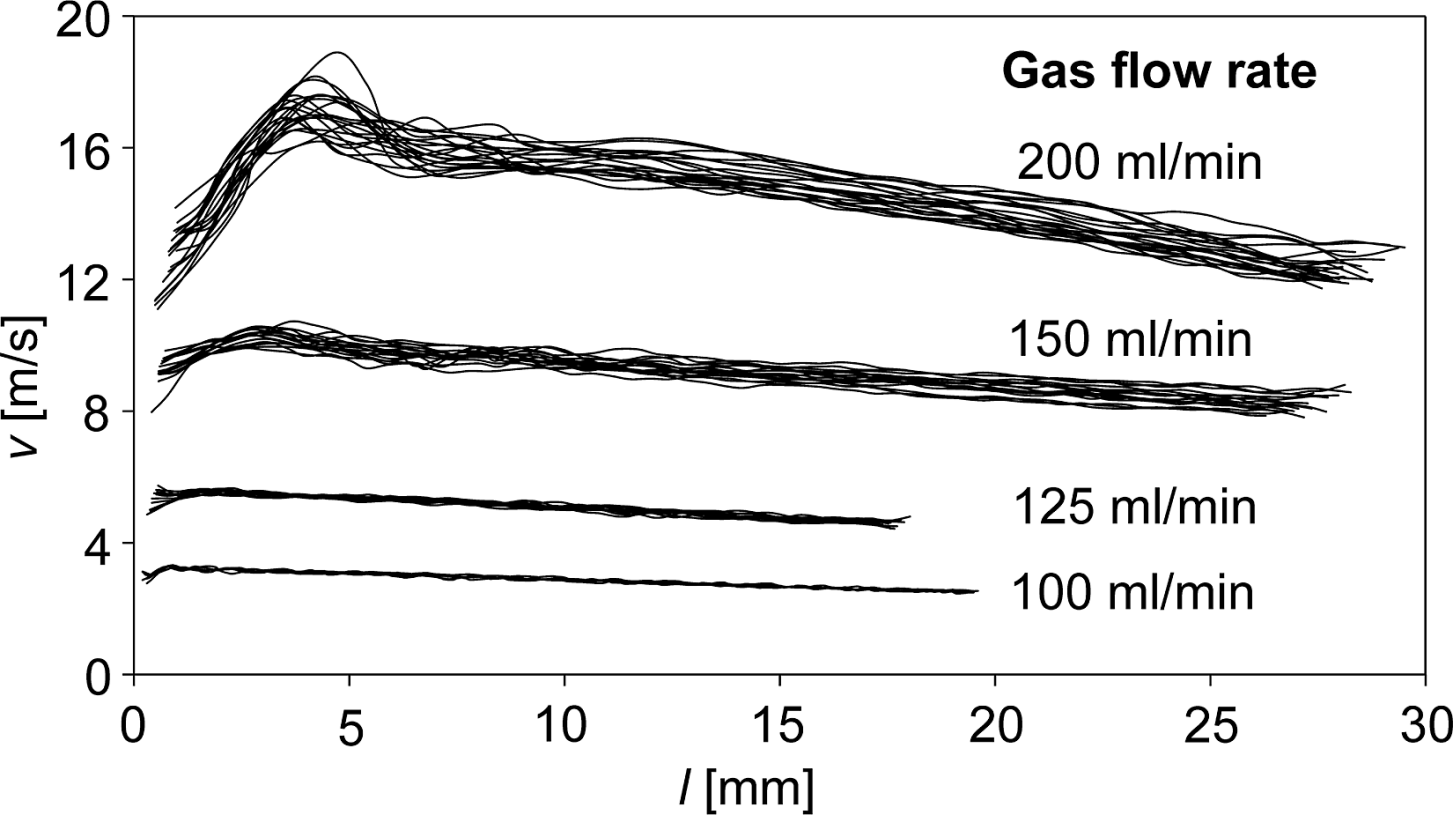
Droplets’ velocity at various distances from the orifice of the nebulizer N5 discharging water at 5.0 μL/min. The dripping regime was achieved at the gas flow rates of 100, 125, 150, and 200 mL/min; velocity profiles of several randomly selected droplets are shown.

The high-speed camera allowed us to also extract data on droplet velocities. As shown in Figure 5, the N5 nebulizer operated in a dripping regime produced droplets exiting the nebulizer orifice at 3–19 m/s. The droplets accelerated up to a distance of several millimeters, and then their velocity gradually decreased. The initial acceleration was due to kinetic energy transferred from the gas molecules to droplets. The speed later decreases because of air resistance. The droplet velocity was greatly affected by the gas flow rate. Smaller droplets generated at higher gas flow rates are lighter and experience larger acceleration. The droplets’ velocity is an important parameter along with the droplet size. Fast-moving droplets may not have enough time to evaporate in the ion;^44^ so, on the other hand, droplets with greater momentum tend to form less-divergent sprays. In ESI, the average velocity of droplets is 2–6 m/s depending on the droplet size, voltage, and spraying mechanism.^41,45^ Thus, the droplets from the GDVN nebulizer in a dripping mode had a comparable velocity to electrospray droplets. However, their size was larger, which could prevent their evaporation during the time they fly in the ion source. The jetting regime produces droplets with higher velocities reaching tens to hundreds of meters per second depending on working parameters.^46,47^ Nevertheless, the jetting mode droplets are very small, which likely permits their evaporation even at short times spent in an ion source.

### Solvents

Compared to ESI, APCI is compatible with a broader range of solvents, including nonpolar ones. The solvent strongly influences ionization processes in APCI, mass spectra, and detection sensitivity and has a major effect on chromatographic separation.^48,49^ The applicability of GDVN was explored for water, 2-propanol, chloroform, methanol, acetonitrile, and toluene. The droplet diameters produced by the N5 nebulizer in a dripping mode at a liquid flow rate of 5.0 μL/min and a gas flow rate of 150 mL/min ranged from 64 to 90 μm. The largest droplets were formed from water, with significantly higher surface tension than other solvents (Table S1). The similar performance, that is, formation of droplets of comparable size from different solvents, promises wide application of the nebulizers in MS and HPLC/MS.

### Geometric Shape of the Spray

To achieve high sensitivity during the ionization step, the diameter of a beam of neutrals produced by a nebulizer should be narrow enough to fit the active ionization zone around the corona discharge (APCI) or UV lamp (APPI). The droplets emerging from a nebulizer tend to create a spray of conical shape. To learn how much the spray spreads over a distance, we performed a simple experiment in which a dye solution was sprayed against a filter paper. The gas flow rate in the N2, N3, and N4 nebulizers was adjusted to achieve a desired jet breakup regime. The diameters of the dye spots were used to reconstruct the spray geometric shape and calculate the spray cone angle Θ (Table 2).

**Table 2:** Spray Angles for Nebulizers N2, N3, and N4 Operated in Dripping and Jetting Regimes at a 1.0 μL/min Liquid Flow Rate^a^

nebulizer	gas flow rate [mL/min]		spray angle Θ [deg]	
	dripping	jetting	dripping	jetting
N2	45	180	1.6 ± 0.3	6.9 ± 0.6
N3	50	190	1.6 ± 0.3	6.8 ± 0.5
N4	70	220	1.2 ± 0.4	6.7 ± 0.3

a The sprayed liquid was Coomassie dye solution in methanol

The nebulizers provided well-focused beams of droplets in both jet breakup regimes. Compared to the dripping mode, the jetting spray spread more rapidly. Still, the spray was narrow, with a spray angle of 6–7°. For N2–N4, the spray angle was almost independent of the exit channel diameter. The narrow spray provided by GDVN nebulizers promises efficient analyte transport into the ionization region of an API source. In contrast to the electrospray, where interdroplet Coulomb repulsion plays a major role, the plume expansion in GDVN is solely driven by hydrodynamic forces.

### GDVN in Micro-APCI Source

**Figure 6:**
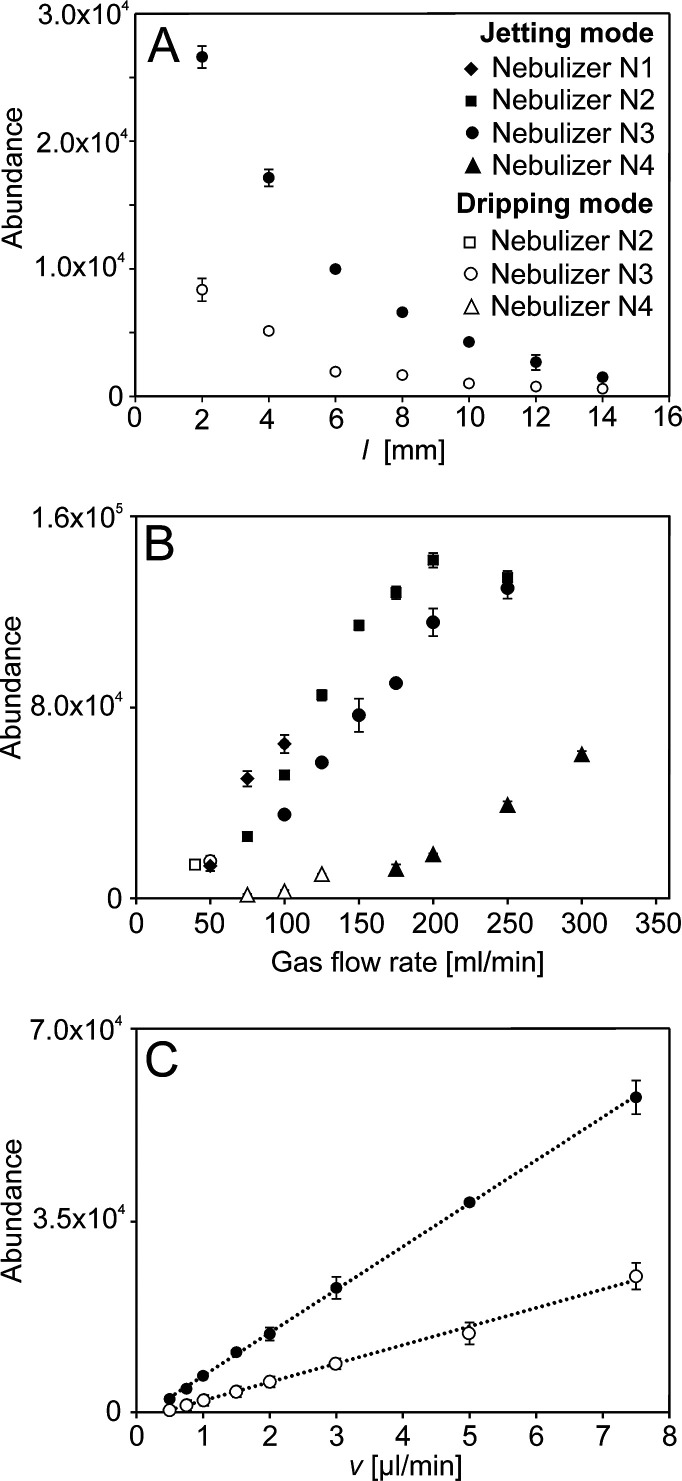
Verapamil peak intensity as a function of the distance between the nebulizer orifice and the corona needle tip (A) and N3 nebulizer discharging toluene solution of verapamil (5.0 μmol/L) at 1.5 μL/min was operated in the dripping mode (gas flow rate of 50 mL/min) or jetting mode (gas flow rate of 175 mL/min). Verapamil peak intensity as a function of the gas flow rate (B) and N1, N2, N3, and N4 nebulizers’ discharging toluene solution of verapamil (5.0 μmol/L) at 1.5 μL/min operated in a dripping or jetting mode depending on the gas flow rate. Verapamil peak intensity as a function of the liquid flow rate (C) and N3 nebulizer discharging toluene solution of verapamil (5.0 μmol/L) was operated in the dripping mode (gas flow rate of 50 mL/min) or jetting mode (gas flow rate of 175 mL/min). The error bars represent standard deviations (*n* = 3).

The nebulizers were employed in an in-house APCI source attached to the LCQ Fleet ion trap mass spectrometer.^21,22,49^ The micro-APCI source geometry was optimized using a verapamil solution (Text S3). The signal of verapamil decreased with the increasing distance between the nebulizer and the discharge electrode tip. As shown for N3 (Figure 6A), the jetting regime provided approximately a 3 times higher signal than the dripping mode in all nebulizer positions. It was explained by smaller droplets formed in the jetting mode; small droplets evaporated quickly, resulting in more efficient transport of analytes from the liquid to the gas phase.

The micro-APCI source geometry was optimized for all nebulizers, and the nebulizers’ performance for verapamil was further tested. Low-intensity and unstable signals were observed for N5, N6, and N7. These nebulizers could only work in a dripping mode producing large droplets that did not vaporize efficiently. The N1, N2, N3, and N4 nebulizers performed much better. The verapamil peak increased with increasing gas flow rate, that is, decreasing droplet diameters (Figure 6B). At lower gas flow rates, the nebulizers operated in the dripping regime. Increasing the gas flow rate led to a change of mode to jetting, which could be observed visually by light scattering on the droplets. Since the droplet size depends on the exit channel diameter, the smallest droplets and hence the highest analyte signal should be observed for N1. However, a very narrow exit channel presented a major pressure restriction that limited the operation of GDVN at higher gas flow rates. In our experiments, the maximum gas flow rate for the N1 nebulizer was 100 mL/min. Therefore, the highest verapamil signals were achieved with N2 and N3 nebulizers having slightly wider exit channels. The verapamil signal intensity increased linearly with increasing sample flow rate for both jet breakup modes of N3 (Figure 6C); the micro-APCI source behaved as a mass-flow sensitive detector, like high-flow rate APCI sources.^21,22,49^

The signal stability was tested for verapamil infused at 4.0 μL/min to the ion source equipped with the N1 or N2 nebulizer. Both nebulizers showed good stability during 10 min recordings; the relative standard deviations for N1 and N2 were 4.7 and 3.8%, respectively. Signal stability did not change dramatically when the liquid flow rate was reduced to 1.5 μL/min (10.0 and 2.7% for N1 and N2, respectively), albeit the spray could be prone to draught-induced instability at very low flow rates^50^ (Figure S4).

Calibration curves allowed us to determine verapamil’s detection limit and dynamic range. The calibration solutions were continuously infused (1.5 μL/min) into the micro-APCI source equipped with the N3 nebulizer. The verapamil signal averaged for 1 min was used to construct calibration curves. A calibration curve was also measured for the conventional heated APCI source. In this case, the verapamil calibration solutions were introduced into the ion source after mixing them in a Tee union with toluene flowing at 1.0 mL/min. This way, the same amount of verapamil per unit of time was delivered into the micro and conventional sources for each calibration solution. For none of the sources, the calibration curves of verapamil were linear (Figure S5). Nevertheless, they allowed us to determine the detection limit as a signal corresponding to 3 times the noise level. As shown in Table 3, the detection limits (amount of verapamil per unit time) were an order of magnitude lower for micro-APCI than the conventional ion source. It should be noted that the microsource was not heated. Aiding droplet evaporation at a higher temperature would likely result in even lower detection limits in micro-APCI sources. The detection limit of verapamil in the micro-APCI source was lower in the jetting mode, which produced smaller droplets. The lower detection limits in the microsource compared to those in the commercial one were attributed to more efficient spray focusing and lower background noise. The reduced background could be explained by significantly less solvent causing chemical noise in the micro-APCI source. Detection limits expressed in the concentration units were lower for conventional APCI than microsources (Table 3), which stems from the nature of mass-sensitive detectors. Even though the micro-APCI sources detected about an order of magnitude less verapamil than the conventional source, the actual concentrations of verapamil in the sprayed solutions were approximately 3 orders of magnitude higher (flow rates of 1.5 μL/min in micro-APCI vs 1.0 mL/min in conventional APCI). In the concentration range tested, the verapamil signal increased with its increasing concentration. Therefore, the upper limit of the dynamic range was not reached even at the highest concentration of verapamil used (50 μmol/L, which corresponds to 1.25 pmol/s).

**Table 3:** Analytical Figures of Merit for Verapamil

	detection limit		dynamic range
ion source	amol/s	[μmol/L]	[amol/s]
micro-APCI (jetting)	42.8	1.71 × 10^–3^	42.8–1.25 × 10^6^^a^
micro-APCI (dripping)	89.0	3.56 × 10^–3^	89.0–1.25 × 10^6^^a^
APCI	875.0	5.25 × 10^–5^	875.0–1.25 × 10^6^^a^

a The highest concentration measured.

The applicability of the micro-APCI source with GDVN for various compounds was studied. The mass spectra of verapamil, acridine, methyl oleate, and palmityl oleate showed abundant protonated molecules (Figure S6). The efficient protonation was enhanced by the open design of the ion source (without housing), which permitted water from the surrounding air to diffuse into the ionization region.^50^ No differences between spectra recorded in the dripping and jetting modes were observed.

## Conclusions

A set of GDVN nebulizers were fabricated and characterized regarding their ability to generate aerosol droplets from liquids delivered at low microliter-per-minute flow rates. The nebulizers were operated either in a dripping regime, producing larger droplets, or jetting regime characterized by smaller droplets. Besides gas and liquid flow rates, the exit channel diameter and the inner tapered capillary-to-orifice distance were important for the nebulizer’s performance. Nebulizers with an exit channel diameter of less than 50 μm worked only in a jetting mode, while nebulizers with a channel diameter larger than 200 μm showed only a dipping mode. Nebulizers with orifice diameters between these values could be operated in either the dripping or jetting mode depending on the gas flow rate settings. While the droplets formed in the dripping regime had tens of micrometers of diameter (45–100 μm), the droplets from the jetting mode were significantly smaller and undetectable by the high-speed camera. The dripping mode droplets left the nebulizer at a speed of several meters per second. Both jet breakup regimes provided a focused spray, with a spray angle of 1–2° for the dripping mode and 6–7° for the jetting mode. The spray was probably narrow enough to fit into the outer edge of the corona, that is, allowed the ionization of analytes in its entire cross-section. The nebulizers worked well for microliter-per-minute flow rates down to 750.0 nL/min; lower flow rates could not be tested because the syringe pump could not reliably push the liquid against high pressure in GDVN.

The GDVN nebulizers were further tested after being mounted in a simple micro-APCI mass spectrometer source. Operating the nebulizers in a jetting mode proved more useful, providing a better response of test analytes than the dripping mode. Complete evaporation of the droplets probably did not occur in the nonheated ion source, even in the jetting mode. Nevertheless, the micro-APCI with GDVN showed strong and stable analyte signals. The micro-APCI source provided an order of magnitude lower detection limit for verapamil (42.8 amol/s) compared to classical heated APCI. The micro-APCI source with GDVN behaved as a mass-flow-sensitive detector and made it possible to ionize a range of compounds. Clogging never occurred during the experiments, indicating the high robustness of the GDVN nebulizers discharging various solutions.

The GDVN nebulizer is useful for nebulizing various solvents and samples at microliter-per-minute flow rates. It can find its use in low-flow-rate APCI and APPI sources, which promise new applications for low- and medium-polar analytes. For routine use, it would be practical to make GDVN nebulizers fixed (nondismantlable), either by using epoxy glue^28^ or by sealing the capillary into the glass of the outer housing. The ion sources with GDVN should be equipped with a heater to evaporate droplets produced by GDVN even more efficiently.

## Supporting Information Available

Additional texts with experimental details, photographs of the nebulizers, spray and micro-APCI source, signal stability data and calibration curves, mass spectra, and diameters of droplets created from various solvents (PDF)
