# Supplementary material for: Gas Dynamic Virtual Nozzle Sprayer for an Introduction of Liquid Samples in Atmospheric Pressure Ionization Mass Spectrometry
Source: Anal Chem. 2023 Feb 17;95(8):4196–203. doi: 10.1021/acs.analchem.2c05349 (PMC10016749; doi:10.1021/acs.analchem.2c05349)
Supplement: Supplementary file 1 — ac2c05349_si_001.pdf [file ac2c05349_si_001.pdf]

Supporting Information

# Gas dynamic virtual nozzle sprayer for an introduction of liquid samples in atmospheric pressure ionization mass spectrometry

Barbora Kloudová[1,2], Timotej Strmeň[1], Vladimír Vrkoslav[1], Zdeněk Chára[3], Ondřej Pačes[1], Josef Cvačka[1,2] *

[1] Institute of Organic Chemistry and Biochemistry of the Czech Academy of Sciences, Flemingovo nám. 2, CZ-166 10 Prague 6, Czech Republic

[2] Department of Analytical Chemistry, Faculty of Science, Charles University in Prague, Hlavova 2030/8, CZ-128 43 Prague 2, Czech Republic

[3] Institute of Hydrodynamics of the Czech Academy of Sciences, Pod Paťankou 30/5, CZ-166 12 Prague 6, Czech Republic

**Text S1**: Fabrication of the nebulizers . . . . . . . . . . S2
**Text S2**: Calculations of the droplet volume . . . . . . . . . . S3
**Text S3**: Optimization of the micro-APCI source . . . . . . . . . . S3
**Text S4**: Chemicals . . . . . . . . . . S4
**Figure S1**: Ion source for micro-APCI. . . . . . . . . . . S4
**Figure S2**: Photographs of the GDVN nebulizers. . . . . . . . . . . S5
**Figure S3**: High-speed camera photographs of the nebulizers. . . . . . . . . . . S5
**Figure S4**: Signal stability for verapamil . . . . . . . . . . S6
**Figure S5**: Calibrations curves for verapamil . . . . . . . . . . S7
**Figure S6**: Mass spectra. . . . . . . . . . . S8
**Table S1**: Solvent properties and the size of droplets . . . . . . . . . . S9

**Text S1: Fabrication of the nebulizers**

Since it has proven difficult to reproducibly fabricate the outer glass housing with a defined exit channel diameter, we tested several fabrication approaches. The initial experiments were performed with laboratory gas and alcohol burners which mostly provided glass housing orifices with poor symmetry along the tube axis and unpredictable exit channel diameter. The glass tube was then fixed in a cordless drill chuck to control heat transfer better. Slow and even rotation (about 600 rpm) helped to improve the orifice shape, but the reproducibility was still limited by flame instability. Replacing the flame with a small, in-house resistance-heated furnace did not help much. The best results were achieved with a micropipette puller allowing precise control of the applied heat. GDVN outer housing units were fabricated using micropipette puller P-100 (Sutter Instrument, Novato, CA) equipped with a 4.5 mm wide box filament. The operating parameters of the micropipette puller were as follows: heat = 900; pull = 0; vel = any positive value; pressure = 500. While heated, the tube was rotated about its axis to fabricate radially symmetric channels. The exit channels of 47 – 320 µm I.D. were obtained by heating the tube for various lengths of time. For instance, about 15 seconds were required to achieve a 50 µm channel. A scanning electron microscope (SEM) Nova NanoSEM 450 (Thermo Fisher Scientific, San Jose, CA) showed a smooth surface and circular shape of the glass tube orifice.

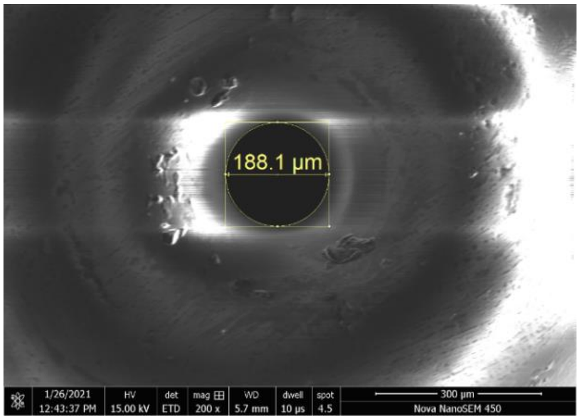


*Electron micrograph; a view into the mouth of the outlet channel in the outer glass housing.*

Since SEM required shortening the tube and thus destroying the outer housing, an optical microscope AM7915MZT from Dino-Lite (Los Angeles, CA) was used for routine determination of the exit channel diameters. The optical microscope was also used during the assembly of the nebulizers. The tapered-tip capillary was centered inside the borosilicate glass housing tube using

an in-house made gas permeable spacer. The spacer made from an aluminum alloy (EN-AW 6060) was a small element of triangular shape with an opening for a 365 µm fused silica capillary.

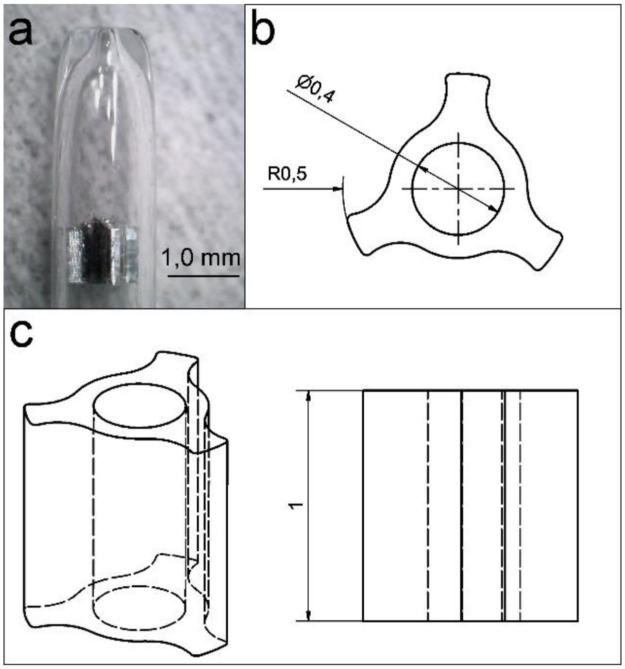


*Photograph of GDVN glass outer housing with the metal spacer (a). Technical drawing of the spacer viewed from the top (b) and side (c). The dimensions are in millimeters.*

## Text S2: Calculations of the droplet volume

The droplet volume was calculated according to the equation $V = v/(60 \times f)$, where $V$ is the volume of the droplet [µl], $v$ is the liquid flow rate [µl/min], and $f$ is the frequency of droplets [$s^{-1}$]. Assuming the spherical shape of a droplet, its diameter $d$ [µm] is given by the formula

$$d = \sqrt[3]{\frac{6 \times 10^9 V}{\pi}}.$$

## Text S3: Optimization of the micro – APCI source

The ion source was assembled on a platform consisting of a flange with two guiding rods. The rods supported a micromanipulator MX10r (Siskiyou, Grant Pass, OR) with the GDVN sprayer and a holder for the corona discharge needle (Figure S1). No heating element was used to aid the solvent evaporation. The ion source geometry was optimized with the N3 nebulizer by infusing verapamil solution (5.0 µmol/l in toluene) into the ion source at a flow rate of 1.5 µl/min. Dripping or jetting mode was achieved by adjusting the gas flow rate to 50 ml/min or 175 ml/min,

respectively. The tip of the corona discharge electrode was positioned 5.0 mm from the mass spectrometry inlet, a distance that guaranteed maximum verapamil signal and no electric sparks. The signal of verapamil decreased with the increasing distance between the nebulizer and the discharge electrode tip. Adjusting the correct position of the nebulizer relative to the mass spectrometer inlet proved easier for the jetting mode, likely due to the larger spray diameter. The nebulizer efficiency, especially in the jetting mode, was high, making it possible to transfer the non-volatile analyte (verapamil) into the gas phase, even without heating. The optimum nebulizer position was 2.0 mm from the discharge needle in both jet breakup regimes.

## Text S4: Chemicals

Methyl and palmityl oleate were purchased from Nu-Chek Prep (Elysian, MN). Acridine (97%) and verapamil (≥99%) were from Sigma-Aldrich (St. Louis, MO) and Coomassie Brilliant Blue G-250 (Imperial™ Protein Stain) from Thermo Fisher Scientific (Waltham, MA). Acetonitrile (LC-MS LiChrosolv®), toluene (Chromasolv® Plus, for HPLC), and 2-propanol (LC-MS Chromasolv®) were obtained from Sigma-Aldrich (St. Louis, MO), and methanol (LC-MS Chromasolv®) and chloroform (for HPLC ≥99.8%) were from Honeywell/Riedel-de Haën (Seelze, Germany). Water was prepared using a Milli-Q integral system (Merck Millipore, Burlington, MA, USA).

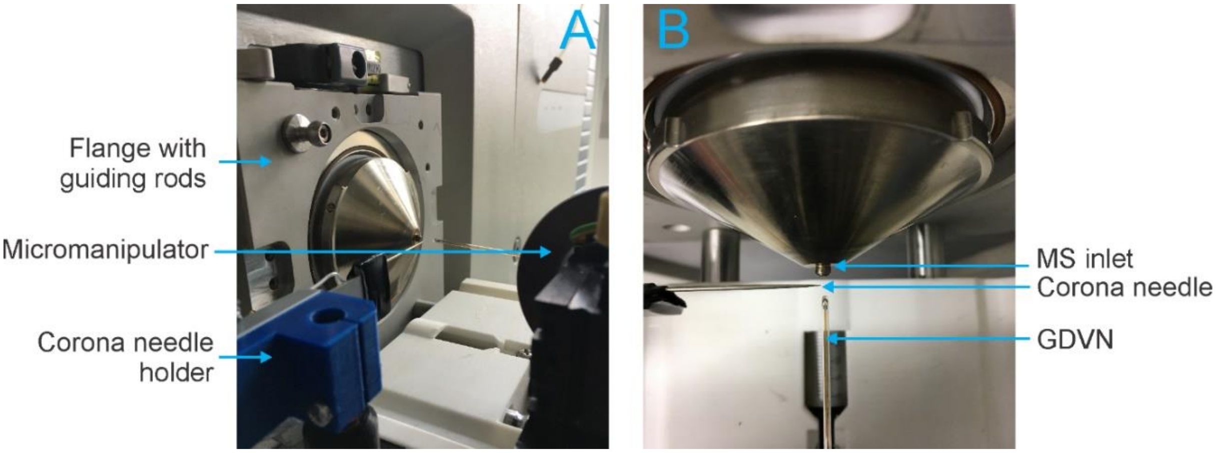


**Figure S1**. Ion source for micro-APCI attached to LCQ Fleet photographed from the side (A) and above (B).

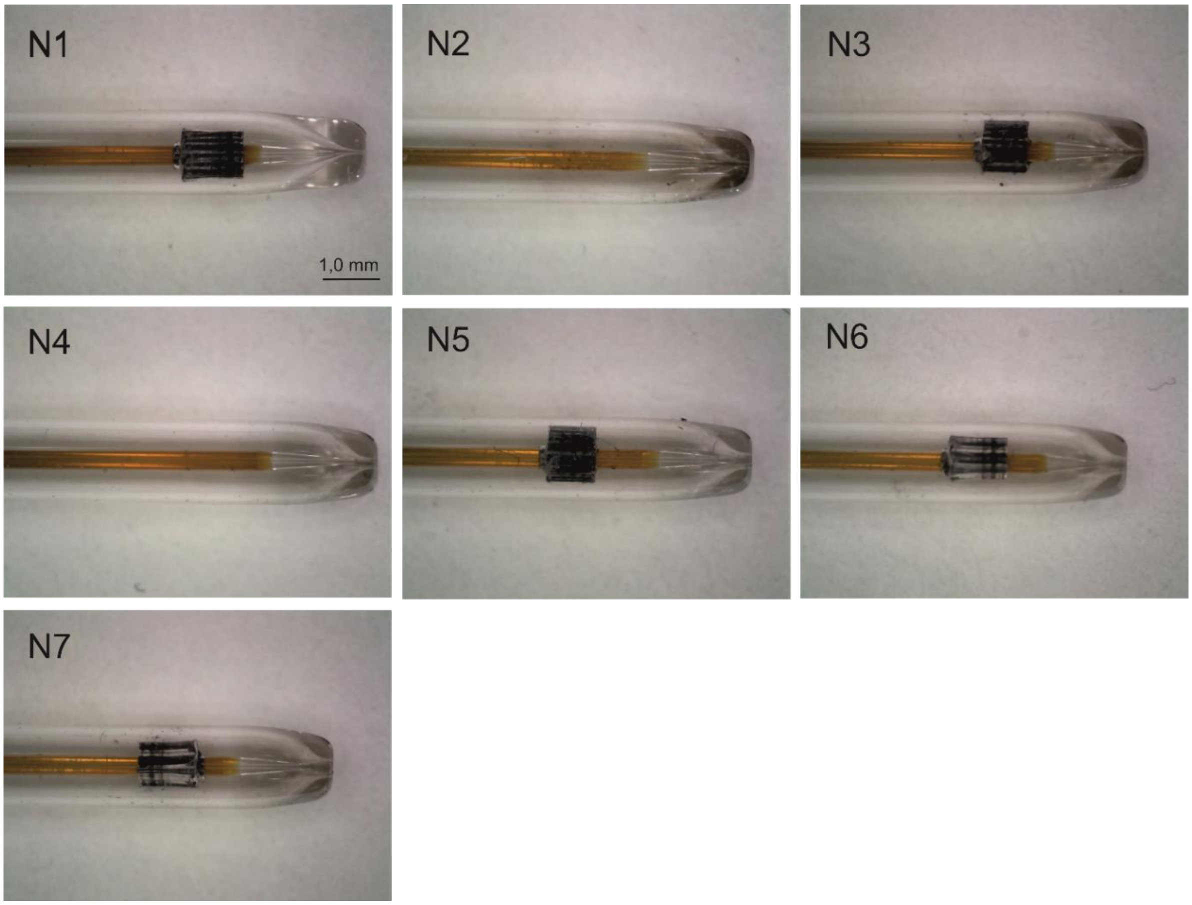


**Figure S2**. Photographs of the GDVN nebulizers captured by Dino-Lite microscope (55× magnification).

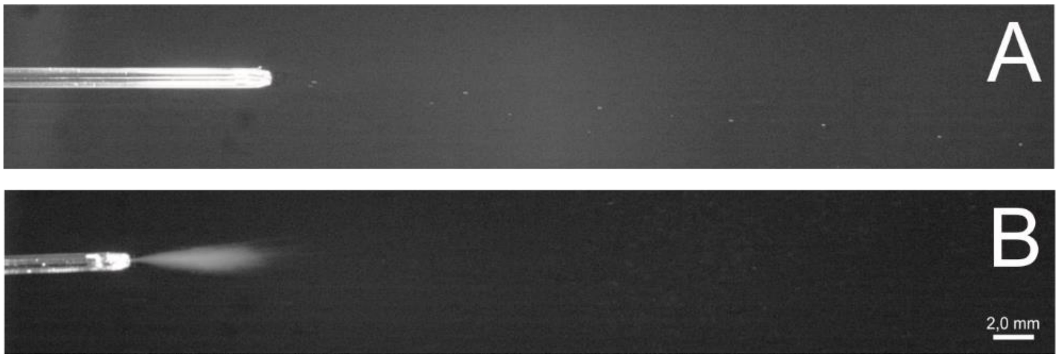


**Figure S3.** High-speed camera photographs of the nebulizers discharging water in the dripping (A; nebulizer N5, liquid flow rate 15.0 μl/min, gas flow rate 150 ml/min) and jetting (B; nebulizer N3, liquid flow rate 10.0 μl/min, gas flow rate 200 ml/min) regimes.

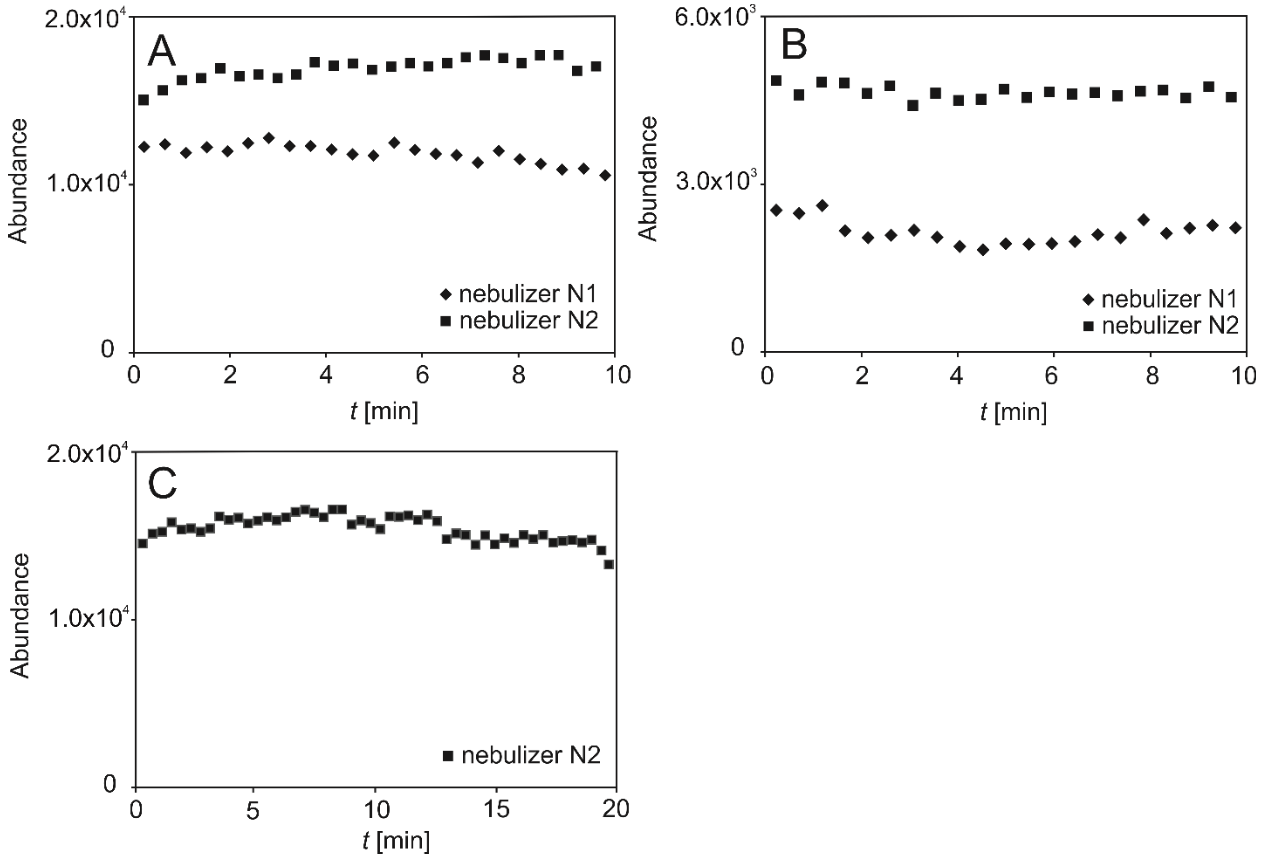


**Figure S4**. Signal stability for verapamil (1.0 µmol/l in toluene) infused at (A) 4.0 µl/min and (B) 1.5 µl/min into the micro-APCI source equipped with N1 (gas flow rate 100 ml/min) and N2 (gas flow rate 100 ml/min). Figure (C) shows 20 min recording of verapamil signal at the same conditions as in (A). The nebulizers operated in the jetting regime. Data points represent intensities of verapamil $[M + H]^+$ peak averaged from 50 scans.

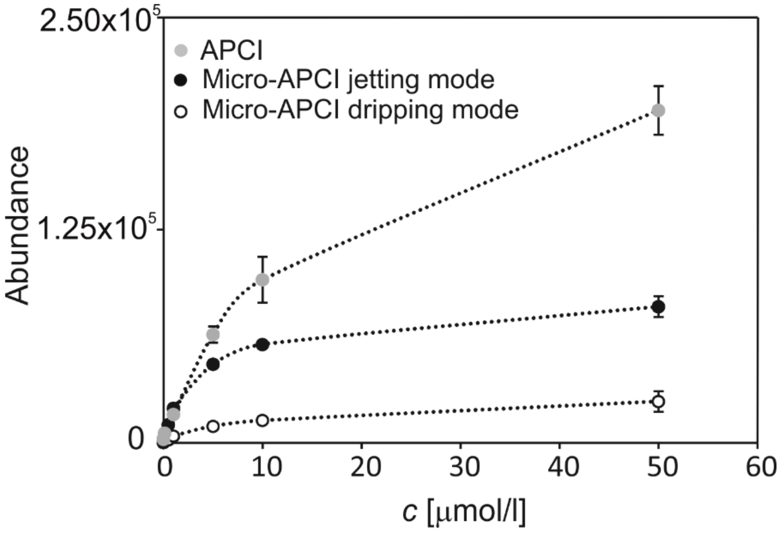


**Figure S5.** Calibrations curves for verapamil infused (1.5 μl/min) into the micro-APCI source (nebulizer N3; gas flow rate of 50 ml/min (dripping mode) or 175 ml/min (jetting mode) and conventional APCI sources. As for the conventional APCI source, the verapamil calibration solutions were introduced into the ion source after mixing them in a Tee union with toluene flowing at 1.0 ml/min.

**Figure S6.** Mass spectra of verapamil (5.0 µmol/l), acridine (5.0 µmol/l), methyl oleate (20.0 µmol/l), and palmityl oleate (10.0 µmol/l) produced by micro-APCI equipped with the N3 nebulizer. The compounds were dissolved in toluene. Spectra were recorded at a sample flow rate of 1.5 µl/min, a gas flow rate of 180 ml/min for jetting mode, and 50 ml/min for dripping mode. All mass spectra were averaged from 1-min records.

**Table S1.**

The physical properties of solvents [SI1-SI3] and the size of droplets produced by the N5 nebulizer in a dripping mode at a liquid flow rate of 5.0 µl/min and gas flow rate of 150 ml/min.

| Solvent | Density [g/ml] | Viscosity [$10^{-3}$ Pa.s] | Surface tension [mN/m] | Droplet diameter* D [µm] |
|---|---|---|---|---|
| Water | 0.998 | 0.89 | 72.80 | 90 ± 1 |
| 2-Propanol | 0.785 | 2.07 | 23.00 | 76 ± 5 |
| Chloroform | 1.498 | 0.54 | 27.50 | 74 ± 2 |
| Methanol | 0.791 | 0.54 | 22.70 | 65 ± 4 |
| Acetonitrile | 0.786 | 0.34 | 29.29 | 64 ± 3 |
| Toluene | 0.867 | 0.55 | 28.40 | 64 ± 1 |

* Mean ± standard deviation ($n$ = 3).

The size of droplets is controlled by a combined effect of the solvents' physico-chemical properties. Viscosity tends to dampen the perturbations and stabilize droplets' growth but has little effect on droplet size[SI4]. In contrast, surface tension affects the droplet volume, hence the time of break up and the shape of the jet. Surface tension increases the cohesion between the fluid elements at the droplet surface and competes against the deformation forces for the system's equilibrium. Therefore, the greater the surface tension, the more fluid comes into the drop, and a bigger drop is formed[SI5]. The droplet size also increases with the liquid density[SI6, SI7].

SI references

SI1. *Properties of Solvents Used in Organic Chemistry*.
http://murov.info/orgsolvents.htm#Chemical_Properties (accessed 2022-10-26).

SI2. *Surface tension values of some common test liquids for surface energy analysis*.
http://www.surface-tension.de/ (accessed 2022-10-26).

SI3. *Surface Tension of Acetonitrile from Dortmund Data Bank*.
http://www.ddbst.com/en/EED/PCP/SFT_C3.php (accessed 2022-11-22).

SI4. Zhang, X.; Basaran, O. A. An Experimental Study of Dynamics of Drop Formation. *Phys. Fluids* **1995**, *7* (6), 1184–1203. https://doi.org/10.1063/1.868577.

SI5. Kadjo, A. Numerical Study of Drop Formation from Dripping and Jetting. Doctoral dissertation, The University of Texas, Arlington, TX, **2011**.

SI6. Mandato, S.; Rondet, E.; Delaplace, G.; Barkouti, A.; Galet, L.; Accart, P.; Ruiz, T.; Cuq, B. Liquids' Atomization with Two Different Nozzles: Modeling of the Effects of Some Processing and Formulation Conditions by Dimensional Analysis. *Powder Technol.* **2012**, *224*, 323–330. https://doi.org/10.1016/j.powtec.2012.03.014.

SI7. Rizkalla, A. A.; Lefebvre, A. H. The Influence of Air and Liquid Properties on Airblast Atomization. *J. Fluids Eng.* **1975**, *97* (3), 316–320. https://doi.org/10.1115/1.3447309.
